# An epidemiologic analysis of the association between eyelid disorders and ocular motility disorders in pediatric age

**DOI:** 10.1038/s41598-022-12883-1

**Published:** 2022-05-25

**Authors:** Matteo Ripa, Giovanni Cuffaro, Pia Clara Pafundi, Paola Valente, Remo Battendieri, Luca Buzzonetti, Roberta Mattei, Stanislao Rizzo, Gustavo Savino

**Affiliations:** 1 Ophthalmology Unit, Fondazione Policlinico Universitario A. Gemelli IRCCS, Rome, Italy; 2 Catholic University of Sacred Heart, Largo Agostino Gemelli, 8, 00168 Rome, Italy; 3 Facility of Epidemiology and Biostatistics, Gemelli Generator, Fondazione Policlinico Universitario A. Gemelli IRCCS, Rome, Italy; 4 Ophthalmology Department, Bambino Gesù IRCCS Pediatric Hospital, Rome, Italy; 5 Consiglio Nazionale delle Ricerche, Istituto di Neuroscienze, Pisa, Italy

**Keywords:** Eyelid diseases, Ocular motility disorders, Vision disorders, Paediatric research

## Abstract

Aim of the study was to assess: (a) the prevalence and type of strabismus, ptosis and eyelid dynamic disorders features, (b) the prevalence of refractive errors, amblyopia and, (c) their association with ocular/systemic syndromes in a cohort of patients. This is a retrospective observational multicenter cohort study. Patients with coexisting ocular motility disorders, comitant and incomitant strabismus, ptosis and dynamic eyelid disorders who have never undergone surgery were enrolled throughout a 3-years a study period. 137 out of 19,089 patients were enrolled, of which 97 with uniocular and 40 with binocular disease. Isolated congenital ptosis was observed in 84 patients. A polymalformative syndrome was present in almost one third of cases, whilst among strabismus type, esotropia was slightly more prevalent. Most patients were hypermetropic. In monocular disease, myopia mainly affected older patients, who were characterized by a worse ptosis margin reflex distance and levator function, and significantly higher astigmatism. Amblyopia occurred in 67.4% of the study sub-population. Of note, in monocular disease this was mild in 25.8%, moderate in 24.2% and severe in 11.3% of cases, whilst in binocular disease it was mild in 25%, moderate in 41.7% and severe in 16.7%. All patients with coexisting eyelid and ocular motility dysfunctions in pediatric age need ophthalmologic and systemic evaluation to accurately assess amblyopia, refractive errors and systemic/ocular disorders.

## Introduction

Static and dynamic eyelid disorders and ocular motility dysfunctions in pediatric age are often related. Strabismus incidence among patients with eyelid disorders has been rarely reported, mainly focused on congenital ptosis. The association between congenital ptosis and hypofunction of the superior rectus muscle commonly occurs in 25% of unilateral forms and in 65% of bilateral forms. Sevel described an embryogenic cause in these patients. During normal fetal development levator muscle and superior rectus are detached by a common epimysium; however, if this does not occur, the mesenchymal tissue portends towards atrophy. This might partly explain why thickened fibrous tissue is often present between the two muscles during attempts to repair congenital ptosis^1^. Griepentrog and Mohney^2^ observed strabismus in 18% of children with ptosis over a 40-years period. Anderson and Baumgartner^3^ reported a prevalence of 36%, of which 48% had vertical squint, and 32% with congenital ptosis, whilst Thapa^4^ of 27% among patients with congenital ptosis. This association may be either an isolated feature or associated to several conditions, such as congenital cranial dysinnervation disorders (CCDD), ocular syndromes (BPTS), orbital diseases, systemic genetic disorders, central nervous system (CNS) disorders and neurological diseases^1–4^. As well, it may also be related, and lead, to refractive errors and amblyopia. Of note, in congenital ptosis a high incidence of astigmatism greater has been observed, with values above 3.50D, which has led many authors to suggest an early examination of refraction in children with ptosis to seek for an appropriate correction^5^. As a matter of fact, in pediatric age, this may significantly affect both visual acuity and normal binocular vision development, mainly involving the eye with either worse ptosis or major refractive error^6^.

Only a few studies have assessed the prevalence of eyelid and ocular motility disorders and their association with functional visual deficits and ocular/systemic syndromes. Hence, the aim of our multicenter retrospective cross-sectional study is to assess, in a cohort of pediatric patients affected by both strabismus and eyelid dynamic disorders, (i) type of strabismus, (ii) prevalence of refractive errors, (iii) functional visual deficits (amblyopia) and (iv) their association with ocular/systemic disorders.

## Materials and methods

### Study design and population

This is a retrospective observational multicenter cohort study, which involved two Italian centers: the Ophthalmology Unit of the Fondazione Policlinico Universitario A. Gemelli IRCCS, Rome, Italy and the Ophthalmology Department of Bambino Gesù IRCCS Pediatric Hospital, Rome.

We included medical records related to all children, aged 6 to16 years old, with coexisting ocular motility disorders, comitant and incomitant strabismus, ptosis and eyelid dynamic disorders either in primary or in other gaze positions, who have never been previously surgically treated, afferent at the two tertiary referral centers from January 1, 2017 through December 31, 2019. All patients with iatrogenic and traumatic eyelid and ocular motility disorders, as well as those previously surgically treated on eyelids or extraocular muscles were instead excluded.

### Data collection and procedures

We collected data from ocular and orthotic examination, including eye motility, cycloplegic refraction, best-corrected visual acuity (BCVA) measurement, upper margin reflex distance (MRD) and levator function (LF) were collected.

Best Correct Visual Acuity (BCVA) was assessed for right and left eye, when possible, using either Lea Symbols charts^7,8^, or ETDRS charts^7^. MRD and LF were measured asking the patient to look at an examination torch held about half a meter away. A ruler was held against the upper eyelid and the distance between the light reflex and superior eyelid margin was measured in primary position (MRD). When patients failed to show a visible light reflex, as their upper lid covered the pupil foramen, either negative or 0 values was assigned, due to analytical purposes. A value of 0 established that the upper lid margin covered the center of the pupil; whereas if equal to − 1, it outstripped the inferior pupil margin. LF was assessed by measuring the excursion in millimeters of the upper eyelid between upgaze and downgaze, fixing the eyebrow.

As for the categories of the analyzed association, these were classified either as “congenital isolated” or “others” (i.e., congenital cranial dysinnervation disorders, genetic diseases, CNS disorders and neurologic diseases, ocular syndromes and orbital diseases). Ptosis features were instead classified either as bilateral and unilateral ptosis or eyelid asymmetry, whereas strabismus as esotropia, exotropia, vertical squint, and either comitant or incomitant. The prevalence of polymalformative syndromes was further assessed.

Ocular alignment was assessed using prism cover-uncover tests. Cover tests were performed with fixation targets both at distance (6 m) and near (33 cm). The refractive status of all patients was instead evaluated by retinoscopy both before and after cycloplegia. Refractive status type (Myopia, Hyperopia, and Astigmatism: with the rule and against the rule) and size (diopters) were recorded. Spherical equivalent (SEq) was calculated as the sum of the spherical plus half of the cylindrical power. Myopia, hyperopia and astigmatism were defined as SEq < 0.5 diopters (D), SEq > 0.5 D and cylindrical error > 1.0 D, respectively.

Amblyopia, where possible, was assessed, according to Pediatric Eye Disease Investigator Group (PEDIG), as mild (20/25 to < 20/40), moderate (20/40 to 20/80) and severe (20/100 to 20/400)^9^. Family history for eyelid abnormalities, oculomotor disorders and systemic/ocular diseases were also recorded.

The study was carried out with the approval from the Fondazione Policlinico Universitario A. Gemelli IRCCS Ethics Committee (Protocol ID number 0039303/20, ID: 3396) and in accordance with 1976 Declaration of Helsinki and its later amendments. A written informed consent for data collection and analysis was obtained from all patients. Photographs were obtained in selected cases previous patients’ permission.

### Statistical analysis

In this retrospective study we examined the charts of all 19.089 children afferent to the two ophthalmologic units involved throughout the three years selected and retrieved 137 patients satisfying the inclusion criteria. Due to retrospective nature we made a post-hoc power calculation based on the primary endpoint of analysing the prevalence of strabismus type (eso- and exo-tropia and vertical squint). Thus, hypothesizing a small to moderate effect size, such sample size achieves 94.0% power to reject the null hypothesis of zero effect size when the population effect size is 0.30 and the significance level (alpha) is 0.050 using a two-sided one-sample z test. Power calculation was performed with PASS 2022 software^10^.

All clinical characteristics were summarized by descriptive statistics techniques. In depth, qualitative variables were expressed by absolute and relative percentage frequencies. Quantitative variables, indeed, were reported either as mean and standard deviation (SD) or median and interquartile range (IQR), as appropriate. Distribution of quantitative data was previously assessed by the Shapiro–Wilk’s test.

Between groups differences for each parameter considered (strabismus type, categories, refractive error, amblyopia, ptosis features) were assessed, as for qualitative variables, either by the Fisher Exact test or the Chi square test, with Yates correction, as appropriate. Quantitative variables were instead assessed either by one-way ANOVA or Student’s t test, whether normally distributed, otherwise either by Mann–Whitney U test or Kruskal–Wallis test. A p value < 0.05 was considered as statistically significant. All analyses were conducted with R software, version 4.1.2 (CRAN^®^, R Core 2021)^11^.

## Results

### General characteristics of the study cohort

During the 3-year study period 137 out of 19.089 patients (0.72%) afferent at the two Ophthalmological Pediatric Centers met the inclusion criteria.

In the same observation period, overall, patients with eyelid static and dynamic disorders were 292 (1.52%) and isolated congenital ptosis were 84 (0.44%).

Ninety-seven out of 137 patients had a monocular disease, affecting either right or left eye, whereas in 40 cases the disease affected both eyes. Patients were also homogeneously distributed for sex (47.4% M vs. 52.6% F), both overall and between groups, with an overall median age of 12 years (IQR 7–16 years).

Among systemic disorders the less prevalent were ocular syndromes and orbital diseases (6%), whilst in almost one third there was a polymalformative syndrome associated (28.5%). Exotropia and Vertical squint types of strabismus were equally distributed (31.4%, n = 43), whilst esotropia was slightly more prevalent (37.2%, n = 51). In addition, most patients were hypermetropic (73.7%, n = 101).

Patients with disease affecting both eyes were characterized by a significantly higher strabismus deviation angle for distance (p = 0.039) and for near (p = 0.023). As well, we observed a significantly higher prevalence of comitant strabismus deviation (p = 0.021) and ptosis familiar history (p = 0.008) in this subgroup. Levator function was instead significantly higher among patients with single-eye disease (median 17 vs 8; p < 0.001). All characteristics of the study population are described in Table 1.

**Table 1:** General characteristics of the study population (n = 137).

	Overall (n = 137)	Monocular disease (n = 97)	Bi-ocular disease (n = 40)	p
Age (years)	12 [7–16]	12 [7–16]	13 [6.5–16]	0.827
**Sex**				0.701
M	65 (47.4)	45 (46.4)	20 (50)	
F	72 (52.6)	52 (53.6)	20 (50)	
**Categories**				**0.004**
Congenital cranial dysinnervation disorders	25 (18.2)	23 (23.7)	2 (5)	
Isolated	27 (19.7)	23 (23.7)	4 (10)	
Genetic	37 (27)	23 (23.7)	14 (35)	
CNS disorders and neurological diseases	38 (27.7)	24 (24.7)	14 (35)	
Ocular syndromes and orbital diseases	10 (7.3)	4 (4.1)	6 (15)	
**Categories**				0.110
Isolated	27 (19.7)	23 (23.7)	4 (10)	
Others	110 (80.3)	74 (76.3)	36 (90)	
Polymalphormative syndromes associated	39 (28.5)	31 (32)	8 (20)	0.158
**Strabismus type**				0.842
Esotropia	51 (37.2)	37 (38.1)	14 (35)	
Exotropia	43 (31.4)	29 (29.9)	14 (35)	
Vertical squint	43 (31.4)	31 (32)	12 (30)	
Strabismus angle deviation distance	14 [6–30]	14 [4–25]	20 [10–30]	**0.039**
Strabismus angle deviation near	14 [6–30]	14 [6–25]	20.5 [10–33.75]	**0.023**
Vertical strabismus	0 [0–5]	0 [0–5]	0 [0–3.75]	0.771
Strabismus onset	2 [1–6]	2 [1–14.3]	1 [1–6]	0.311
Strabismus familiar history	7 (5.1)	3 (3.1)	4 (10)	0.214
**Strabismus deviation type**				**0.021**
Comitant	75 (54.7)	47 (48.5)	28 (70)	
Incomitant	62 (45.3)	50 (51.5)	12 (30)	
Ptosis familiar history	12 (8.8)	4 (4.1)	8 (20)	**0.008**
**Ptosis features**				** < 0.001**
Bilateral ptosis	34 (24.8)	–	34 (85)	
Bilateral eyelid	6 (4.4)	–	6 (15)	
Asymmetry unilateral ptosis	67 (48.9)	67 (69.1)	–	
Unilateral eyelid asymmetry	30 (21.9)	30 (30.9)	–	
Ptosis MRD (mm)	3 [1–4]	3 [2–4]	1 [1–3]	** < 0.001**
Ptosis onset	2 [1–6]	2 [1–6]	1 [1–6]	0.715
Levator function	16 [10–18]	17 [14–18]	8 [4.25–15]	** < 0.001**
**Refractive error**				0.658
Myopic	34 (24.8)	24 (24.7)	10 (25)	
Hypermetropic	101 (73.7)	71 (73.2)	30 (75)	
Anisometropic	2 (1.5)	2 (2.1)	–	
Refractive error	2.25 [− 0.5 to 3]	2.25 [− 0.5 to 3.13]	2.25 [− 0.38 to 2.94]	0.589
Amblyopia*				0.187
None	28 (32.6)	24 (38.7)	4 (16.7)	
Mild	22 (25.6)	16 (25.8)	6 (25)	
Moderate	25 (29.1)	15 (24.2)	10 (41.7)	
Severe	11 (11.8)	7 (11.3)	4 (16.7)	
**Fixing eye**				0.581
Right	60 (43.8)	44 (45.4)	16 (40)	
Left	44 (32.1)	32 (33)	12 (30)	
Alternative fixation	33 (24.1)	21 (21.6)	12 (30)	
Is astigmatism evaluable?	79 (57.7)	57 (58.8)	22 (55)	0.685
Astigmatism value	0.5 [0–1]	0.5 [0–1]	0.5 [0–2]	0.517
Is BCVA evaluable?	85 (62)	61 (62.9)	24 (60)	0.752
BCVA ptosis	0.1 [0–0.45]	[0–0.35]	0.3 [0.1–0.68]	**0.040**

*M* male, *F* female, *CNS* central nervous system, *MRD* margin reflex distance, *BCVA* best corrected visual acuity.

*Others: congenital cranial dysinnervation disorders, Genetic, CNS disorders/neurological diseases, Ocular syndromes/orbital diseases.

Significant data are in bold, suggestive p-values in italic.

Each outcome of interest was further separately compared between the two subgroups.

### Strabismus

Strabismus was classified as follows: esotropia, exotropia and vertical squint.

Patients with monocular disease showed that ptosis onset occurs significantly later in those with vertical squint (median 6 months vs.1 in exotropic and esotropic patients, p = 0.010). A suggestive association instead emerged as for what concerns strabismus onset (p = 0.083), towards a later occurrence in those with vertical squint.

For what concerns ptosis features, all patients with vertical squint had unilateral ptosis, with the congenital ones significantly more prevalent (83.9% vs. 51.4% in esotropia and 75.9% in exotropia, respectively; p = 0.010).

Other relevant findings are related to the refractive error value, significantly higher in those with esotropia (median 2.5 vs 1.75 in exotropia and 2 in vertical squint, respectively; p = 0.044), whilst BCVA was less evaluable in patients with esotropia (48.6% vs, 65.5% vs. 77.4%, respectively; p = 0.047).

Findings were similar also in patients with binocular disease as for vertical strabismus (median 18 months in those with vertical squint vs. 0 in both other strabismus types, p < 0.001), whilst ptosis familiar history was significantly prevalent in those with exotropia (42.9% vs. 14.3 in esotropia and 0 in vertical squint, p = 0.019). A suggestive association instead emerged towards a younger age (median 10 years vs. 16 years in exotropia and 13 years in vertical squint; p = 0.073) and higher MRD values (3 mm vs 1 mm in the other two categories; p = 0.081) among patients with esotropia. All data are reported in Table 2.

**Table 2:** Association between strabismus type and general and clinical characteristics in the study cohort, according to patients’ subgroups (n = 137).

	Monocular disease (n = 97)				Binocular disease (n = 40)			
	Esotropia (n = 37)	Exotropia (n = 29)	Vertical squint (n = 31)	p	Esotropia (n = 14)	Exotropia (n = 14)	Vertical squint (n = 12)	p
Age (years)	12 [7–16]	10 [7–16]	12 [10–15]	0.577	10 [6–14]	16 [3–17]	13 [12–17]	*0.073*
**Sex**				0.713				0.123
M	19 (51.4)	12 (41.4)	14 (45.2)		10 (71.4)	6 (42.9)	4 (33.3)	
F	18 (48.6)	17 (58.6)	17 (54.8)		4 (28.6)	8 (57.1)	8 (66.7)	
**Categories**				0.353				0.433
Isolated	6 (16.2)	9 (31)	8 (25.8)		2 (14.3)	0 (0.0)	2 (16.7)	
Others*	31 (83.8)	20 (69)	23 (74.2)		12 (85.7)	14 (100)	10 (83.3)	
Polymalphormative syndromes associated	13 (35.1)	9 (31)	9 (29)	0.858	2 (14.3)	2 (14.3)	4 (33.3)	0.427
Strabismus angle deviation distance	15 [5–22.5]	12 [5–27.5]	12 [0–25]	0.660	20 [10–25]	20 [8–30]	20 [10–35]	0.936
Strabismus angle deviation near	14 [6–25]	14 [7–19]	10 [0–20]	0.296	25 [10–30]	15 [8–30]	23 [10–55]	0.676
Vertical strabismus	0 [0–0]	0 [0–0]	10 [5–16]	** < 0.001**	0 [0–0]	0 [0–0]	18 [5–40]	** < 0.001**
Strabismus onset	2 [1–6]	2 [1–34.8]	6 [1–48]	*0.083*	1 [1–6]	1 [1–108]	4 [1–6]	0.746
Strabismus familiar history	–	2 (6.9)	1 (3.2)	0.275	2 (14.3)	2 (14.3)	0 (0.0)	0.524
Ptosis familiar history	1 (2.7)	2 (6.9)	1 (3.2)	0.665	2 (14.3)	6 (42.9)	0 (0.0)	**0.019**
**Strabismus deviation type**				0.181				0.265
Concomitant	17 (45.9)	18 (62.1)	12 (38.7)		12 (85.7)	8 (57.1)	8 (66.7)	
Incomitant	20 (54.1)	11 (37.9)	19 (61.3)		2 (14.3)	6 (42.9)	4 (33.3)	
**Ptosis features**				**0.010**				0.182
Bilateral ptosis	–	–	–		10 (71.4)	12 (85.7)	12 (100)	
Bilateral eyelid asymmetry	–	–	–		4 (28.6)	2 (14.3)	0 (0.0)	
Unilateral ptosis	19 (51.4)	22 (75.9)	26 (83.9)		–	–	–	
Unilateral eyelid asymmetry	18 (48.6)	7 (24.1)	5 (16.1)		–	–	–	
Ptosis MRD (mm)	3 [2–4]	3 [2–4]	3 [1–4]	0.121	3 [1–4]	1 [1–3]	1 [1–2]	*0.081*
Ptosis onset	1 [1–4]	2 [1–17.3]	6 [1–60]	**0.010**	1 [1–6]	1 [1–108]	4 [1–6]	0.750
Levator function	18 [15–18.5]	16 [12–18]	18 [14–18]	0.489	15 [3–16]	8 [4–14]	8 [5–14]	0.709
**Refractive error**				0.242				0.811
Myopic	5 (13.5)	8 (27.6)	11 (35.5)		4 (28.6)	4 (28.6)	2 (16.7)	
Hypermetropic	31 (83.8)	21 (72.4)	19 (61.3)		10 (71.4)	10 (71.4)	10 (83.3)	
Anisometropic	1 (2.7)	–	1 (3.2)		0 (0.0)	0 (0.0)	0 (0.0)	
Refractive error	2.5 [2 to 4]	1.75 [− 0.13 to 3]	2 [− 1 to 3]	**0.044**	2.55 [− 4.00 to 7.00]	1.50 [− 2.00 to 2.75]	1.75 [1.0 to 2.5]	0.391
Amblyopia**				0.597				-
None	6 (31.6)	6 (31.6)	12 (50)		2 (25.0)	2 (25.0)	0 (0.0)	
Mild	7 (36.8)	4 (21.1)	5 (20.8)		0 (0.0)	2 (25.0)	4 (50.0)	
Moderate	5 (26.3)	6 (31.6)	4 (16.7)		6 (75.0)	4 (50.0)	0 (0.0)	
Severe	1 (5.3)	3 (15.8)	3 (12.5)		0 (0.0)	0 (0.0)	4 (50.0)	
**Fixing eye**				0.949				**0.045**
Right	17 (45.9)	12 (41.4)	15 (48.4)		10 (71.4)	2 (14.3)	4 (33.3)	
Left	11 (29.7)	11 (37.9)	10 (32.3)		2 (14.3)	6 (42.9)	4 (33.3)	
Alternate fixation	9 (24.3)	6 (20.7)	6 (19.4)		2 (14.3)	6 (42.9)	4 (33.3)	
Is astigmatism evaluable?	18 (48.6)	19 (65.5)	20 (64.5)	0.282	10 (71.4)	6 (42.9)	6 (50.0)	0.289
Astigmatism value	0.25 [0–1]	0.5 [0–1]	0.75 [0–1]	0.726	1.75 [1.00–2.00]	1.00 [1.00–3.00]	1.75 [0.5–2.5]	0.880
Is BCVA evaluable?	18 (48.6)	19 (65.5)	24 (77.4)	**0.047**	8 (57.1)	8 (57.1)	8 (66.7)	0.848
BCVA ptosis	0.1 [0–0.3]	0.2 [0–0.4]	0.05 [0–0.38]	0.487	0.45 [0.15–0.60]	0.20 [0.05–0.55]	0.45 [0.1–0.95]	0.790

*M* male, *F* female, *MRD* margin reflex distance, *BCVA* best corrected visual acuity.

*Others: congenital cranial dysinnervation disorders, Genetic, CNS disorders/neurological diseases, Ocular syndromes/orbital diseases.

**Amblyopia was analysed on the subgroup of patients for which information was available.

Significant data are in bold, suggestive p-values in italic.

### Ptosis features

We thus analysed patients according to ptosis features, i.e. congenital ptosis or eyelid asymmetry, either unilateral or bilateral, according to the mono- or bin-ocular disease.

In patients with monocular disease, vertical strabismus emerged as significantly higher among those with unilateral congenital ptosis (p = 0.011). Moreover, we observed a significantly higher prevalence of esotropia in patients with unilateral eyelid asymmetry (60% vs. 28.4%; p = 0.010). In the same subgroup, also MRD (median 4 mm vs. 3 mm; p < 0.001) and levator function (median 18 mm vs. 16 mm; p < 0.001) were significantly higher than among those with congenital ptosis.

Looking at patients with both eyes affected, findings were almost similar, with MRD (median 4 mm vs. 1 mm; p < 0.001) and LF (median 18 mm vs. 8 mm; p < 0.001) both significantly higher in patients with bilateral eyelid asymmetry as compared to congenital. Conversely, astigmatism value was significantly lower in this subset of patients (median 0.75 vs. 2; p = 0.026). A suggestive association instead emerged towards a slightly higher refractive error among those with eyelid asymmetry (p = 0.060), and towards a higher strabismus deviation angle for near in those with congenital ptosis (p = 0.093). All data are reported in Table 3.

**Table 3:** Association between ptosis features and general and clinical characteristics in the study cohort, according to patients’ subgroups (n = 137).

	Monocular disease (n = 97)			Binocular disease (n = 40)		
	Unilateral congenital ptosis (n = 67)	Unilateral eyelid asymmetry (n = 30)	p	Bilateral congenital ptosis (n = 34)	Bilateral eyelid asymmetry (n = 6)	p
Age (years)	12 [8–16]	10 [7–15]	0.159	13 [6–16]	14 [10–16]	0.648
**Sex**			0.398			0.661
M	33 (49.3)	12 (40)		16 (47.1)	4 (66.7)	
F	34 (50.7)	18 (60)		18 (52.9)	2 (33.3)	
**Categories**			0.275			1.000
Isolated	18 (26.9)	5 (16.7)		4 (11.8)	–	
Others*	49 (73.1)	25 (83.3)		30 (88.2)	6 (100)	
Polymalphormative syndromes associated	24 (35.8)	7 (23.3)	0.223	6 (17.6)	2 (33.3)	0.580
**Strabismus type**			**0.010**			0.182
Esotropia	19 (28.4)	18 (60)		10 (29.4)	4 (66.7)	
Exotropia	22 (32.8)	7 (23.3)		12 (35.3)	2 (33.3)	
Vertical squint	26 (38.8)	5 (16.7)		12 (35.3)	0 (0.0)	
Strabismus angle deviation distance	12 [4–25]	14 [5.5–20]	0.888	25 [10–30]	10 [6–16]	**0.047**
Strabismus angle deviation near	14 [6–25]	13 [6–21.3]	0.876	30 [10–35]	10 [8–16]	*0.093*
Vertical strabismus	0 [0–8]	0 [0–0]	**0.011**	0 [0–5]	0 [0–0]	0.135
Strabismus onset	2 [1–30]	2 [1–6]	0.240	1 [1–6]	1 [1–108]	0.933
Strabismus familiar history	2 (3)	1 (3.3)	0.927	4 (11.8)	0 (0.0)	1.000
**Strabismus deviation type**			0.500			0.153
Comitant	34 (50.7)	13 (43.3)		22 (64.7)	6 (100)	
Incomitant	33 (49.3)	17 (56.7)		12 (35.3)	0 (0.0)	
Ptosis familiar history	4 (6)	–	0.172	8 (23.5)	0 (0.0)	0.318
Ptosis MRD (mm)	3 [2–3]	4 [3–4]	** < 0.001**	1 [1–2]	4 [4–4]	** < 0.001**
Ptosis onset	2 [1–13]	1.5 [1–6]	0.310	1 [1–6]	1 [1–108]	0.800
Levator function	16 [12–18]	18 [17.8–19]	** < 0.001**	8 [4–14]	18 [16–18]	** < 0.001**
**Refractive error**			0.457			0.307
Myopic	18 (26.9)	6 (20)		10 (29.4)	0 (0.0)	
Hypermetropic	47 (70.1)	24 (80)		24 (70.6)	6 (100)	
Anisometropic	2 (3)	–		0 (0.0)	0 (0.0)	
Refractive error	2.25 [− 0.5 to 3]	2.25 [1 to 3.25]	0.504	1.625 [− 0.50 to 2.75]	2.55 [2.25 to 6.50]	*0.060*
Amblyopia**			0.699			n.a.
None	16 (37.2)	8 (42.1)		0 (0.0)	4 (100)	
Mild	10 (23.3)	6 (31.6)		6 (30.0)	0 (0.0)	
Moderate	11 (25.6)	4 (21.1)		10 (50.0)	0 (0.0)	
Severe	6 (14)	1 (5.3)		4 (20.0)	0 (0.0)	
**Fixing eye**			0.167			0.102
Right	33 (49.3)	11 (36.7)		14 (41.2)	2 (33.3)	
Left	23 (34.3)	9 (30)		8 (23.5)	4 (66.7)	
Alternate fixation	11 (16.4)	10 (33.3)		12 (35.3)	0 (0.0)	
Is astigmatism evaluable?	41 (61.2)	16 (53.3)	0.467	18 (52.9)	4 (66.7)	0.673
Astigmatism value	0.5 [0–1]	0.4 [0–1]	0.523	2.0 [1.0–3.0]	0.75 [0.5–1.0]	**0.026**
Is BCVA evaluable?	43 (64.2)	18 (60)	0.694	20 (58.8)	4 (66.7)	1.000
BCVA ptosis, median [IQR]	0.1 [0–0.4]	0.1 [0–0.3]	0.624	0. 5 [0.1–0.7]	–	n.a.

*M* male, *F* female, *MRD* margin reflex distance, *BCVA* best corrected visual acuity, *n.a.* not applicable.

*Others: congenital cranial dysinnervation disorders, Genetic, CNS disorders/neurological diseases, Ocular syndromes/orbital diseases.

**Amblyopia was analysed on the subgroup of patients for which information was available.

Significant data are in bold, suggestive p-values in italic.

### Refractive errors

Refractive error was classified as myopic, hypermetropic and anisometropic.

In patients with monocular disease, patients were significantly older among those with myopia (median 14.5 years vs. 10 hypermetropic and 11 anisometropic, p = 0.005). Moreover, refractive error was significantly lower in myopic subjects, though such a result has to be confirmed due to the too small sample of anisometropic patients.

For what concerns patients with binocular disease, instead, patients were only either myopic or hypermetropic. In this subgroup of patients, strabismus onset occurred significantly later in myopic subjects (median 6 months vs. 1, p < 0.001). A comparable finding was also observed for ptosis onset. Myopic subjects were also characterized by a significantly higher prevalence of ptosis familiar history (60% vs. 6.7%, p = 0.001) and higher astigmatism value (median 3 vs. 1, p = 0.003), and a lower refractive error (median -3 vs. 2.5, p < 0.001). Conversely, both MRD (median 2 mm vs.1 mm, p = 0.003) and LF (median 14 mm vs. 3 mm, p < 0.001) were significantly higher among hypermetropic patients. All data are shown in Table 4.

**Table 4:** Association between type of refractive error and general and clinical characteristics in the study cohort, according to patients’ subgroups (n = 137).

	Monocular				Binocular		
	Myopic (n = 24)	Hypermetropic (n = 71)	Anisometropic (n = 2)	p	Myopic (n = 10)	Hypermetropic (n = 30)	p
Age (years)	14.5 [12–17]	10 [7–15]	11 [7–/]	**0.005**	17 [10–17]	13 [6–16]	0.117
**Sex**				0.865			0.465
M	10 (41.7)	34 (47.9)	1 (50)		6 (60.0)	14 (46.7)	
F	14 (58.3)	37 (52.1)	1 (50)		4 (40.0)	16 (53.3)	
**Categories**				**0.043**			0.556
Isolated	5 (20.8)	18 (25.4)	–		0 (0.0)	4 (13.3)	
Others	19 (79.2)	53 (74.6)	2 (100)		10 (100)	26 (86.7)	
Polymalphormative syndromes associated	6 (25)	24 (33.8)	1 (50)	0.623	0 (0.0)	8 (26.7)	0.165
**Strabismus type**				0.242			0.811
Esotropia	5 (20.8)	31 (43.7)	1 (50)		4 (40.0)	10 (33.3)	
Exotropia	8 (33.3)	21 (29.6)	–		4 (40.0)	10 (33.3)	
Vertical squint	11 (45.8)	19 (26.8)	1 (50)		2 (20.0)	10 (33.3)	
Strabismus angle deviation distance	16.5 [7–30]	12 [4–20]	21 [12–/]	0.270	25 [25–30]	10 [8–30]	0.024
Strabismus angle deviation near	15 [7–25]	12 [4–25]	19 [8–/]	0.450	30 [25–30]	10 [8–35]	*0.068*
Vertical strabismus	0 [0–9]	0 [0–2]	2.5 [0–/]	0.296	0 [0–0]	0 [0–5]	0.805
Strabismus onset	2.5 [1–70.3]	2 [1–6]	3.5 [1–/]	0.537	6 [6–14]	1 [1–6]	** < 0.001**
Strabismus familiar history	–	3 (4.2)	–	0.567	0 (0.0)	4 (13.3)	0.556
**Strabismus deviation type**				0.352			0.451
Comitant	11 (45.8)	36 (50.7)	–		6 (60.0)	22 (73.3)	
Incomitant	13 (54.2)	35 (49.3)	2 (100)		4 (40.0)	8 (26.7)	
Ptosis familiar history	1 (4.2)	3 (4.2)	–	0.957	6 (60.0)	2 (6.7)	**0.001**
**Ptosis features**				0.457			0.307
Bilateral ptosis	–	–	–		10 (100)	24 (80.0)	
Bilateral eyelid asymmetry	–	–	–		0 (0.0)	6 (20.0)	
Unilateral ptosis	18 (75)	47 (66.2)	2 (100)		–	–	
Unilateral eyelid asymmetry	6 (25)	24 (33.8)	–		–	–	
Ptosis MRD (mm)	3 [2–4]	3 [2–4]	2 [1–/]	0.561	1 [0–1]	2 [1–4]	**0.003**
Ptosis onset	2 [1–68.4]	2 [1–6]	3.5 [1–/]	0.483	6 [6–138]	1 [1–6]	** < 0.001**
Levator function	18 [16–18]	16 [14–18]	18 [18–18]	0.293	3 [3–4]	14 [7–16]	** < 0.001**
Refractive error	− 1 [− 2.25 to − 0.5]	2.75 [2 to 3.5]	− 6.5 [− 11 to /]	** < 0.001**	− 3 [− 4 to − 2]	2.50 [1.75 to 3.25]	** < 0.001**
**Amblyopia**				0.141			–
None	9 (45)	15 (36.6)	–		0 (0.0)	4 (28.6)	
Mild	4 (20)	15 (36.6)	–		0 (0.0)	6 (42.9)	
Moderate	4 (20)	11 (26.8)	–		8 (80.0)	2 (14.3)	
Severe	3 (15)	3 (7.3)	1 (50)		2 (20.0)	2 (14.3)	
**Fixing eye**				0.500			0.603
Right	8 (33.3)	35 (49.3)	1 (50)		4 (40.0)	12 (40.0)	
Left	11 (45.8)	20 (28.2)	1 (50)		4 (40.0)	8 (26.7)	
Alternate fixation	5 (20.8)	16 (22.5)	–		2 (20.0)	10 (33.3)	
Is astigmatism evaluable? n (%)	14 (58.3)	41 (57.7)	2 (100)	0.488	6 (60.0)	16 (53.3)	1.000
Astigmatism value	0.4 [0–1.2]	0.5 [0–1]	1.75 [1.5–/]	0.158	3 [2–3]	1.00 [0.75–1.75]	**0.003**
Is BCVA evaluable? n (%)	20 (83.3)	40 (56.3)	1 (50)	*0.057*	10 (100)	14 (46.7)	**0.003**
BCVA ptosis	0.1 [0–0.4]	0.1 [0–0.3]	1 [1–1]	0.263	0.6 [0.6–0.7]	0.1 [0.0–0.3]	0.125

*M* male, *F* female, *MRD* margin reflex distance, *BCVA* best corrected visual acuity, *IQR* interquartile range.

Significant data are in bold, suggestive p-values in italic.

### Diseases categories

For what concerns categories, we considered all categories grouped vs. the isolated one, in order to minimize the bias due to small sample sizes of the single subgroups.

Looking first at monocular disease, we observed a significantly higher prevalence of associated polymalformative syndromes in the isolated category (37.8% vs. 13%, p = 0.026). A suggestive association instead emerged towards a higher prevalence of incomitant strabismus deviation type (56.8% vs. 34.8%, p = 0.066) and a higher mild amblyopia (50% vs. 17.4%, p = 0.060) in patients with other diseases.

Among patients with binocular disease, indeed, in the isolated category there was a significantly higher prevalence of strabismus familiar history (50% vs. 5.6%, p = 0.043), and a suggestive association towards a higher MRD (median 3 mm vs. 1 mm, p = 0.064). All data are reported in Table 5.

**Table 5:** Association between disease categories and general and clinical characteristics in the study cohort, according to patients’ subgroups (n = 137).

	Monocular			Binocular		
	Other (n = 74)	Isolated (n = 24)	p	Isolated (n = 4)	Others (n = 36)	p
Age (years)	12 [7.8–16]	12 [7–15]	0.683	10 [6–13]	14 [8–16]	0.319
**Sex**			0.524			1.000
M	33 (44.6)	12 (52.2)		2 (50.0)	18 (50.0)	
F	41 (55.4)	11 (47.8)		2 (50.0)	18 (50.0)	
Polymalphormative syndromes associated	28 (37.8)	3 (13)	**0.026**	–	8 (22.2)	0.566
**Strabismus type**			0.353			0.433
Esotropia	31 (41.9)	6 (26.1)		2 (50.0)	12 (33.3)	
Exotropia	20 (27)	9 (39.1)		–	14 (38.9)	
Vertical squint	23 (31.1)	8 (34.8)		2 (50.0)	10 (27.8)	
Strabismus angle deviation distance	13 [4–26.3]	14 [6–20]	0.812	17 [4–30]	20 [10–30]	0.467
Strabismus angle deviation near	12 [4–25]	16 [8–25]	0.278	20 [4–35]	21 [10–30]	0.585
Vertical strabismus	0 [0–5]	0 [0–12]	0.417	0 [0–0]	0 [0–5]	0.236
Strabismus onset, mean (SD)	2 [1–17]	2 [1–13]	0.919	6 [6–6]	1 [1–6]	0.108
Strabismus familiar history	1 (1.4)	2 (8.7)	0.277	2 (50.0)	2 (5.6)	**0.043**
**Strabismus deviation type**			*0.066*			0.297
Comitant	32 (43.2)	15 (65.2)		4 (100)	24 (66.7)	
Incomitant	42 (56.8)	8 (34.8)		–	12 (33.3)	
Ptosis familiar history	4 (5.4)	–	0.590	–	8 (22.2)	0.566
**Ptosis features**			0.275			1.000
Bilateral ptosis	–	–		4 (100)	30 (83.3)	
Bilateral eyelid asymmetry	–	–		–	6 (16.7)	
Unilateral ptosis	49 (66.2)	18 (78.3)		–	–	
Unilateral eyelid asymmetry	25 (33.8)	5 (21.7)		–	–	
Ptosis MRD (mm)	3 [2–4]	3 [2–4]	0.968	3.0 [2.5–3.5]	1.0 [1.0–3.0]	*0.064*
Ptosis onset	2 [1–9.9]	2 [1–6]	0.405	6 [6–6]	1 [1–6]	0.108
Levator function	18 [14–18]	16 [15–18]	0.608	15 [14–15]	8 [4–16]	0.221
**Refractive error**			0.658			0.556
Myopic	19 (25.7)	5 (21.7)		–	10 (27.8)	
Hypermetropic	53 (71.6)	18 (78.3)		4 (100)	26 (72.2)	
Anisometropic	2 (2.7)	–		–	–	
Refractive error	2 [− 0.5 to 3]	3 [1 to 3.25]	0.185	2 [1.75 to 2.25]	2.25 [− 0.5 to 3.125]	0.928
Amblyopia*			*0.060*			n.a.
None	21 (45.7)	3 (18.8)		–	4 (16.7)	
Mild	8 (17.4)	8 (50)		–	6 (25.0)	
Moderate	12 (26.1)	3 (18.8)		–	10 (41.7)	
Severe	5 (10.9)	2 (12.5)		–	4 (16.7)	
**Fixing eye**			0.733			**0.031**
Right	34 (45.9)	10 (43.5)		4 (100)	12 (33.3)	
Left	23 (31.1)	9 (39.1)		–	12 (33.3)	
Alternative fixation	17 (23)	4 (17.4)		–	12 (33.3)	
Is astigmatism evaluable? n (%)	43 (58.1)	14 (60.9)	0.814	2 (50.0)	20 (55.6)	1.000
Astigmatism value	0.5 [0–1]	0.5 [0–1]	0.989	0.5 [0.5–0.5]	1.75 [1.0–2.75]	n.a.
Is BCVA evaluable? n (%)	45 (60.8)	16 (69.6)	0.448	–	24 (66.7)	**0.020**
BCVA Ptosis	0.1 [0–0.4]	0.2 [0.1–0.3]	0.306	–	0.3 [0.1–0.7]	n.a.

*M* male, *F* female, *MRD* margin reflex distance, *BCVA* best corrected visual acuity.

*Amblyopia was analysed on the subgroup of patients for which information was available.

Significant data are in bold, suggestive p-values in italic.

### Amblyopia

Sixty-two patients with monocular disease were assessed for the visual acuity vs. 24 with binocular disease. Fifty-one uncooperative patients (37.2%) were not assessed. Amblyopia was reported in 58 out of 86 patients (67.4%).

Thirty-eight out of 62 patients with monocular disease were diagnosed as amblyopic (61.3%), according to Pediatric Eye Disease Investigator Group (PEDIG), of which 16 (25.8%) were diagnosed as mild, 15 (24.2%) as moderate and 7 (11.3%) as severe. Twenty-four patients (38.7%) did not show amblyopia in the affected eye.

Among the 24 patients with binocular disease assessed, amblyopia was diagnosed in 60% of cases. Six patients (25.0%) were diagnosed as mild, 10 (41.7%) as moderate and 4 (16.7%) as severe, whilst the remaining four patients (16.7%) did not have amblyopia.

Moreover, 33.3% of the patients with moderate amblyopia and monocular disease presented polymalformative syndromes. All data are reported in Table 6.

**Table 6:** Association between amblyopia and general and clinical characteristics in the study cohort according to patients’ subgroups.

Monocular disease	Amblyopia			
	Absent (n = 24)	Mild (n = 16)	Moderate (n = 15)	Severe (n = 7)
Age (years)	12 [8.5–16]	12.5 [8.3–18]	11 [8–14]	12 [12–15]
**Sex**				
M	8 (33.3)	6 (37.5)	8 (53.3)	1 (28.6)
F	16 (66.7)	10 (62.5)	7 (46.7)	5 (71.4)
**Categories**				
Isolated	6 (25)	5 (31.3)	4 (26.7)	–
Others*	18 (75)	11 (68.7)	11 (73.3)	7 (100)
Polymalphormative syndromes	–	4 (25)	5 (33.3)	3 (42.9)
**Strabismus type**				
Esotropia	6 (25)	7 (43.8)	5 (33.3)	1 (14.3)
Exotropia	6 (25)	4 (25)	6 (40)	3 (42.9)
Vertical squint	12 (50)	5 (31.3)	4 (26.7)	3 (42.9)
Strabismus angle deviation distance	12 [0–17.5]	16 [8–20]	15 [4–35]	20 [6–30]
Strabismus angle deviation near	8 [4–15.5]	14 [7–23.3]	20 [8–35]	12 [6–25]
Vertical strabismus	1 [0–8]	0 [0–9]	0 [0–0]	0 [0–5]
Strabismus onset	4.5 [1–48]	4 [1–110.9]	4 [1–30]	6 [1–21.5]
Strabismus familiar history	3 (12.5)	–	–	–
**Strabismus deviation type**				
Comitant	9 (37.5)	8 (50)	9 (60)	4 (57.1)
Incomitant	15 (62.5)	8 (50)	6 (40)	3 (42.9)
**Ptosis features**				
Bilateral ptosis	–	–	–	–
Bilateral eyelid asymmetry	–	–	–	–
Unilateral ptosis	16 (66.7)	10 (62.5)	11 (73.3)	6 (85.7)
Unilateral eyelid asymmetry	8 (33.3)	6 (37.5)	4 (26.7)	1 (14.3)
Ptosis familiar history	–	–	1 (6.7)	1 (14.3)
Ptosis MRD (mm)*	3 [3–4]	3.5 [2.3–4]	3 [2–4]	2 [1–4]
Ptosis onset	3.5 [1–57]	2 [1–6]	4 [2–6]	6 [1–21.5]
Levator function	18 [16.3–18.8]	18 [16–18]	16 [14–18]	16 [10–18]
**Refractive error**				
Myopic	9 (37.5)	4 (25)	4 (26.7)	3 (42.9)
Hypermetropic	15 (62.5)	12 (75)	11 (73.3)	3 (42.9)
Anisometropic	–	–	–	1 (14.2)
Refractive error	1.4 [− 0.5 to 2.9]	2.8 [− 0.1 to 3.4]	2.5 [− 1.25 to 3.25]	− 0.5 [− 7.5 to 2.5]
**Fixing eye**				
Right	11 (45.8)	5 (31.3)	11 (73.3)	4 (57.1)
Left	8 (33.3)	8 (50)	1 (6.7)	3 (42.9)
Alternative fixation	5 (20.8)	3 (18.8)	3 (20)	–
Is astigmatism evaluable?	15 (62.5)	10 (62.5)	9 (60)	5 (71.4)
Astigmatism value	0.5 [0–1]	0.63 [0–1]	0.5 [0–1.25]	1 [0–1]
Is BCVA evaluable?	23 (95.8)	16 (100)	15 (100)	7 (100)
BCVA ptosis	0 [0–0]	0.1 [0.1–0.2]	0.4 [0.3–0.5]	1 [1–1]

Binocular disease	Absent (n = 4)	Mild (n = 6)	Moderate (n = 10)	Severe (n = 4)
Age (years)	13 [10–16]	13 [12–16]	15 [10–17]	18 [17–18]
**Sex**				
M	2 (50)	4 (66.7)	6 (60)	–
F	2 (50)	2 (33.3)	4 (40)	4 (100)
**Categories**				
Isolated	–	–	–	–
Others*	4 (100)	6 (100)	10 (100)	4 (100)
Polymalphormative syndromes association	–	2 (100)	–	–
**Strabismus type**				
Esotropia	2 (50)	–	6 (60)	–
Exotropia	2 (50)	2 (33.3)	4 (40)	–
Vertical squint	0	4 (66.7)	–	4 (100)
Strabismus angle deviation distance	8 [6–10]	30 [10–35]	25 [25–30]	35 [0–70]
Strabismus angle deviation near	9 [8–10]	40 [10–55]	30 [25–30]	35 [0–70]
Vertical strabismus	0 [0–0]	5 [0–50]	0 [0–0]	35 [30–40]
Strabismus onset	55 [1–108]	1 [1–6]	6 [6–6]	7 [1–14]
Strabismus familiar history	–	–	–	–
**Strabismus deviation type**				
Comitant	4 (100)	4 (66.7)	6 (60)	–
Incomitant	–	2 (33.3)	4 (40)	4 (100)
**Ptosis features**				
Bilateral ptosis	–	6 (100)	10 (100)	4 (100)
Bilateral eyelid asymmetry	4 (100)	–	–	–
Unilateral ptosis	–	–	–	–
Unilateral eyelid asymmetry	–	–	–	–
Ptosis familiar history	–	–	6 (60)	–
Ptosis MRD (mm)*	4.0 [3.5–4.5]	1.0 [1.0–3.0]	1.0 [0.0–1.0]	1.0 [0.5–1.5]
Ptosis onset	55 [1–108]	1 [1–6]	6 [6]	69 [1–138]
Levator function	17 [16–18]	14 [5–17]	3 [3–4]	6 [2–9]
**Refractive error**				
Myopic	–	–	8 (80)	2 (50)
Hypermetropic	4 (100)	6 (100)	2 (20)	2 (50)
Anisometropic	–	–	–	–
Refractive error	2.4 [2.3 to 2.6]	1.0 [0.0 to 1.5]	− 3.0 [− 4.0 to − 2.0]	1.0 [− 0.5 to 2.5]
**Fixing eye**				
Right	2 (50)	2 (33.3)	4 (40)	2 (50)
Left	2 (50)	–	2 (20)	2 (50)
Alternative fixation	–	4 (66.7)	4 (40)	–
Is astigmatism evaluable?	2 (50)	2 (33.3)	8 (80)	2 (50)
Astigmatism value	0.75 [0.5–1.0]	2.5 [2.5–2.5]	2.50 [1.75–3.00]	2.0 [1.0–3.0]
Is BCVA evaluable?	4 (100)	6 (100)	10 (100)	4 (100)
BCVA ptosis	0 [0–0]	0.1 [0.1–0.1]	0.6 [0.3–0.6]	0.95 [0.85–1]

Amblyopia was analysed on the subgroup of patients for which information was available.

Qualitative variables are expressed as absolute and percentage frequency quantitative data as median and interquartile range (IQR).

*M* male, *F* female, *MRD* margin reflex distance, *BCVA* best corrected visual acuity.

*Others: congenital cranial dysinnervation disorders Genetic CNS disorders/neurological diseases ocular syndromes/orbital diseases.

## Discussion

Over a 3-year study period, this multicenter study analyzed 137 out of 19.089 patients (0.72%) afferent at the two Ophthalmological Pediatric Centers. The overall prevalence of isolated congenital ptosis was 0.44% (84 out of 19,089 patients), of whom 27 (32.1%) were associated with ocular motility disorders.

The reported prevalence of strabismus associated with congenital ptosis ranges from 18 to 36%^1–4^, remarkably higher than in the general population^12^. In about one third there was a concomitant diagnosis of polymalformative syndrome. The binocular type shows a significantly higher prevalence of ptosis familiar history, comitant strabismus, higher strabismus deviation angle and lower levator function values.

Regarding strabismus type (esotropia, exotropia, vertical squint), congenital unilateral ptosis was more prevalent in patients with vertical squint. Some Authors suggested that strabismus could be secondary to a disruption of binocularity by the ptotic eyelid^13^. On the other hand, as for congenital ptosis associated with vertical strabismus, a prenatal insult or maldevelopment of the third cranial nerve might play an important role^14,15^. Moreover, consistent with the current literature, refractive error value was significantly higher in patients with esotropia^16^.

As for ptosis features, we observed a significantly higher prevalence of esotropia and mild ptosis, as well as a good levator function in patients with either unilateral or bilateral eyelid asymmetry. These data are mainly related to the clinical features of Type I Duane Retraction Syndrome^17^.

With regard to refractive error features, patients with monocular disease were significantly older in the myopic subgroup, and they also had a significantly worse ptosis MRD and LF, as well as higher astigmatism values. Several authors reported more severe refractive errors in either moderate or severe ptosis. Zeng et al. reported a higher frequency of hyperopia and a lower axial length–corneal radius (AL/CR) ratio in case of severe ptosis, thus suggesting a delay in the growth of the eye^18^. Huo et al. instead deemed that long-standing congenital ptosis might produce myopia^19^. The refractive modifications were also related to a mechanic effect of the ptotic eyelid on ocular surface which reshapes the corneal surface^20,21^.

Of note, this study presents several limitations, mainly due to the retrospective nature of the study. This has surely affected inclusion of parameters which might have been interesting to be investigated, such as genes and mutations involved in these patients. However, genetic data were not available for all patients, as they are not routine examination. We hope to include such an important issue in future prospective studies on this topic. In addition, the small sample size did not allow to deeply examine each feature, which was in turn characterized by more sub-categories. The concomitant presence of ocular motility disorders and eyelid dynamic disorders establishes a rare condition, which would need the co-participation of several referral centres for a targeted study.

Finally, our data, though limited by the small sample size, confirm a mechanical and deprivation effect of moderate and severe eyelid ptosis, which may lead to refractive changes.

The monocular isolated association, as compared with the other categories, is significantly related to polymalformative syndromes (37.8% vs. 13%, p = 0.026), and more often shows an incomitant strabismus deviation.

Amblyopia occurred in the 67.4% of the assessed subpopulation. In monocular disease the 25.8% was diagnosed as mild, the 24.2% as moderate and 11.3% as severe (vs. the 25%, 41.7% and 16.7% in those with binocular disease). Amblyopia estimated prevalence in the general population ranges from the 2.5% to 5.6%^22,23^*.* The reported prevalence of amblyopia in patients with ptosis, and both ptosis and strabismus, is 7–8 times higher, and it is associated to a higher prevalence of refractive errors, as well as to a not alternating strabismus^6,12,24,25^*.*

## Conclusions

To the best of our knowledge, this is the first study investigating the relation of eyelids and ocular motility disorders. Eyelid static and dynamic disorders and ocular motility disorders are often related. Ocular motility evaluation and follow-up is especially important in congenital ptosis, where the ptotic lid may in fact precipitate strabismus and amblyopia. The high incidence of polymalformative syndromes in monocular disease should alert the specialist to a possible multidisciplinary assessment. Ultimately these patients thus need a careful ophthalmologic and systemic evaluation, due the high prevalence of amblyopia, refractive errors and systemic/ocular associated disorders.

## Data Availability

The datasets generated and/or analyzed during the current study are not publicly available due the common policy of our institution. However, they are available from the corresponding author on reasonable request.
